# Influence of Emergence Angle and Mucosal Tunnel Depth on Artificial Biofilm Removal Around Dental Implants: An In Vitro Study

**DOI:** 10.1155/ijod/7500003

**Published:** 2025-11-11

**Authors:** Nicola Discepoli, Isabella De Rubertis, Delia David, Alice Ferrari, Raffaele Mirra

**Affiliations:** Unit of Periodontics, Department of Medical Biotechnologies, Università degli Studi di Siena, Siena, Italy

**Keywords:** angle, dental abutments, dental implant-abutment design, dental implants, dental prosthesis design, prevention

## Abstract

**Background:**

Implant-prosthetic characteristics jeopardize accurate diagnosis, professional and domiciliary plaque control around dental implants. Accurate prosthetic design planning and prosthetic features modifications are fundamental in peri-implant diseases' primordial prevention and active treatment.

**Objectives:**

To evaluate the impact of prosthetic emergence angles (EAs) and mucosal tunnel depths (MTDs) on the efficacy of ultrasonic debridement in removing ink stain simulating artificial biofilm in an in vitro model.

**Methods:**

An in vitro model simulating biofilm around implant abutment, incorporating a 4 mm implant analog replicating a missing single tooth was designed. Titanium abutments with three MTDs (2, 4, and 6 mm) were associated with individualized crowns with different EAs (15°, 30°, and 45°), resulting in nine experimental groups. Abutments were stained with artificial biofilm and subsequently instrumented through ultrasonic debridement. The proportion of residual biofilm (ResB) was quantified and evaluated for the four surfaces.

**Results:**

A total of 360 images of 90 instrumented abutments was evaluated. The overall means described a consistent increase of ResB in relation to the progressive increment of both MTD and EA. Mesial and distal surfaces presented more biofilm across all EA-MTD combinations (*p*  < 0.05). Logistic regression models pinpointed MTD and EA as significant predictors. The 6 mm MTD and 45° EA combination demonstrated as the strongest predictor (odds ratio [OR] = 134,33).

**Conclusions:**

The combination of a progressively wider prosthetic EA and a deeper mucosal tunnel significantly reduced the efficacy of submucosal instrumentation. Narrower EA (<30°) and shallower MTD (<4 mm) yielded significantly better results in terms of ResB.

## 1. Introduction

Over the last decades, the rise in the global number of individuals receiving dental implants as restorative therapy has turned peri-implant health into a major issue. Findings on the prevalence of peri-implant diseases reported a subject-based prevalence of 43% as far as it concerns for peri-implant mucositis (PIM) and of 22% for peri-implantitis [1, 2]. Hence, the recently updated Clinical Practice Guideline on the prevention and treatment of peri-implant diseases [3] revised the definition of peri-implant biological complications, aligning it with the modifications introduced by the ID-COSM initiative consensus [4]. Clinical signs of PIM account for more than one bleeding point around the implant (or a line or profuse bleeding at any location), with or without suppuration, and in the absence of bone loss, beyond the initial crestal bone level changes.

Promptly addressing and effectively managing PIM is crucial to prevent its progression to peri-implantitis and to restore a condition compatible with peri-implant health [5, 6].

Although the reversibility of PIM has been demonstrated [7, 8], recent clinical studies have shown that, regardless of the treatment modality and despite observable clinical improvements, none of the currently available protocols consistently achieve complete disease resolution [9–11]. A systematic review [12] investigated nonsurgical therapies for PIM and underpinned that no single method demonstrated clinical superiority. Indeed, it remains unclear whether combinations of different procedures or repeated treatments might offer additional benefits. In this perspective, implant-prosthetic design features might be considered crucial to support both self-performed oral hygiene and an accurate clinical diagnosis [13–15]. Available evidence suggests that prosthetic characteristics, as the emergence angle (EA) and the mucosal tunnel depth (MTD), negatively impact short-term treatment outcomes and influence long-term peri-implant tissues health [14, 16–18]. Katafuchi et al. [18] demonstrated that both an EA greater than 30° and a convex prosthetic profile are notable risk indicators for peri-implantitis, thus, modifying the prosthetic profile becomes a critical supplement to nonsurgical treatment in cases where oral hygiene accessibility is restricted [14, 15, 17]. Moreover, a recent preclinical study demonstrated that wide restorative angles are associated with more plaque accumulation and an increased risk of marginal bone loss compared to narrow angles [19]. Similarly, deep mucosal tunnel has been shown to decrease the effectiveness of peri-implant diseases treatment [20] and to compromise the resolution of mucosal inflammation [21]. While modifying a suboptimal prosthetic should be provided as part of nonsurgical periodontal implant therapy [3], the ability to alter the mucosal tunnel through nonsurgical therapy is virtually negligible. This underscores the importance of primordial prevention, starting from the treatment plan stage [3].

The abundance of confounding factors that typically characterize clinical cases makes it particularly challenging to isolate specific elements related to therapeutic efficacy. In this context, the creation of an artificial controlled environment may prove valuable, as it allows for the establishment of proof-of-concept studies that can inform and direct future clinical research. Therefore, the objective of the present in vitro study is to analyze the impact of different prosthetic EAs and MTDs on the effectiveness of ultrasonic debridement in artificial biofilm removal. The secondary objective is to determine the optimal combination of EA and MTD for maximizing professional mechanical plaque removal efficacy.

## 2. Materials and Methods

### 2.1. In Vitro Model Design

The present investigation is reported in accordance with the Checklist for Reporting In-vitro Studies (CRIS) guidelines [22].

The in vitro model was developed based on an impression of a patient missing the upper right first molar, rehabilitated with a single dental implant. The model, devoid of supporting bone loss, included a 4 mm implant analog (B&B Dental, Argelato [BO], Italy). Three different titanium abutments measuring 2, 4, and 6 mm in height (B&B Dental, Argelato [BO], Italy) were selected and used in the present in vitro study. The abutment height was measured from the implant shoulder to the most coronal point of the abutment. The MTD, assessed as the linear distance between the implant shoulder and the coronal margin of the peri-implant mucosa, corresponded to the height of the experimental abutments [21].

**Figure 1:**
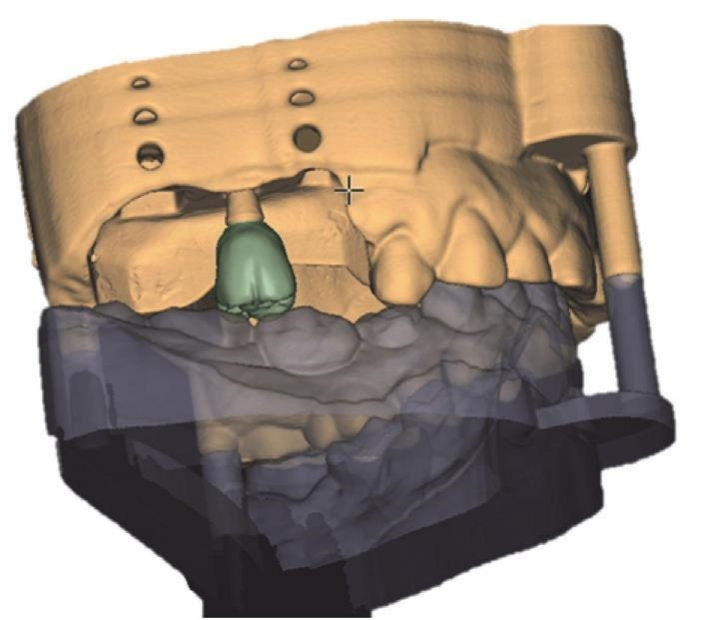
Crown realization-making process with Exocad Dental DB 3.1 Software.

Prosthetic crowns were fabricated from polymethyl methacrylate (PMMA) using Exocad Dental DB 3.1 software (Figure 1) and specifically designed to meet the experimental requirements with three distinct EAs: 15°, 30°, and 45°. The prosthetic EAs have been chosen according to the available literature [23–25], defining 15° and 45° as thresholds values. For each abutment height under study (2, 4, and 6 mm), three crowns were created, one per EA, resulting in a total of three crowns per abutment height. Each crown was customized to fit only the specific abutment height it was designed for. The crown was seated onto the designated abutment, secured, and then removed only after the experimental phase. For each abutment height, a customized peri-implant mucosa (gingival mask [GM]) was initially sculpted in wax. Subsequently, it was replicated using a light-body polyvinylsiloxane material (Gingifast Soft, Zhermack, Marl, Germany) to closely simulate natural mucosal anatomy, as previously described [26]. A new GM replica was produced for each instrumentation to maintain consistent experimental conditions, which could be influenced by wear of the polyvinylsiloxane after use.

### 2.2. Experimental Phase

Nine experimental groups were established based on the different combinations of MTD(2, 4, and 6 mm) and EAs (15°, 30°, and 45°).

A single independent operator (AF) was responsible for marking the transmucosal surfaces of the abutments with indelible ink (Staedtler permanent Lumocolor, Nürnberg, Germany) to simulate the presence of dental biofilm and for assembling the model (GM, abutment, and prosthetic crown). The transmucosal abutment surface was covered with an even application of indelible ink by means of a permanent marker, making a single layer application for the whole surface. After each instrumentation session, the same operator unscrewed the abutments, cleaned them with an air rinse, and prepared the model for the next session. The sequence of EA-MTD combinations was determined using a computer-generated randomized order. Additionally, the same operator took standardized digital photographs of each of the four abutment surfaces (vestibular, oral, mesial, and distal) using a Nikon D3500 camera (Tokyo, Japan) in a light box with ISO 800, f/18, a shutter speed of 1/50, and a distance of 11 cm. A distinctive feature of the colored ink remnants is their high visibility, which enables investigators to accurately identify the surface areas unaffected by the ultrasonic device.

**Figure 2:**
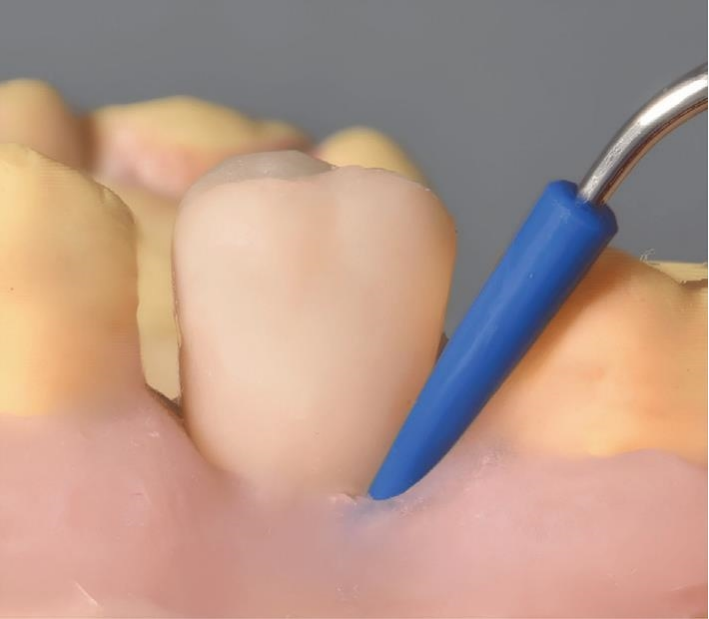
Simulation of intervention on the peri-implant artificial biofilm model.

A single trained periodontist (IDR), masked to the EA-MTD combination under study, performed the ultrasonic debridement. The submucosal debridement was carried out using a magnetostrictive device (Cavitron Select SPS, Dentsply Sirona) equipped with fine ultrasonic tips and a silicone insert (SlimLine 30 k insert, SofTip implant 30 k insert, Dentsply) (Figure 2).

To ensure consistency in the treatment, the instrumentation time was standardized at 120 s per experimental unit, corresponding to 20 s per site (vestibular, oral, vestibulomesial, vestibulodistal, disto-oral, and mesio-oral). The procedure was carried out using short, horizontal strokes in a mesiodistal direction, starting from a randomly assigned surface and progressing clockwise. Throughout the procedure, the ultrasonic tip was gently advanced to the most apical point allowed by the model.

### 2.3. Residual Biofilm (ResB) Assessment

The digital images obtained were analyzed by a trained and calibrated examiner (DD), who was not involved in the in vitro experimentation. Using image processing software (ImageJ 1.52a, U.S. National Institutes of Health, Bethesda, MD, USA; https://imagej.nih.gov/ij/), the examiner identified ink-stained areas to determine the percentage of ResB.

The entire transmucosal abutment surface was considered as the region of interest (ROI), and the abutment boundaries were manually outlined. The red color was selected as the threshold, and the hue, saturation, and brightness (HSB) color model were applied. The brightness and saturation were manually adjusted until the detected area precisely matched the ink-stained regions (Figure S1).

The amount of ResB on each of the four surfaces—vestibular, oral, mesial and distal—was then calculated as [26]: area of ResB (pixels)/area of the test surface (pixels) × 100 (%).

### 2.4. Sample Size Calculation

Sample size calculation was based on criteria from a previous study [26], which observed a posttreatment ResB percentage of 32% following ultrasonic debridement. To ensure a study power of 80% with an alpha error of 0.05, a total of 90 abutments were included in the present investigation (command “power,*”* STATA IC, version 18, StataCorp LP, TX, USA).

### 2.5. Statistical Analysis

A dedicated statistical software was used to conduct the analysis (STATA IC, version 18, StataCorp LP, TX, USA). Data were analyzed considering the abutment as the experimental unit. Additionally, subgroup analyses were performed according to surface (vestibular, oral, mesial, and distal), EA (15°, 30°, and 45°), and MTD (2, 4, and 6 mm). Continuous data were presented as mean and standard deviation (SD). The Shapiro-Wilk test was run to assess the normal data distribution, while homogeneity of variances was tested using Levene's test. To account for potential intraunit correlation, subgroup analyses were conducted independently for each surface type, treating the abutment as the unit of analysis, and avoiding pooling all surface measurements into a single model. Intra- and intergroup comparisons based on MTD, EA, and surface were analyzed using one-way analysis of variance (ANOVA), followed by Bonferroni-corrected post hoc tests. Statistical significance was set at *p*  < 0.05. Finally, a logistic regression model was built to assess the impact of MTD, EAs, and their interaction (independent variables) on the likelihood of ResB retention (dependent variable). The continuous variable ResB was dichotomized based on its median value (0.31) and used as binary independent variable. Values ≥ 0.31 were classified as “high residual biofilm,” while values < 0.31 were classified as “low residual biofilm.” The best model was chosen according to the lowest values of Akaike information criteria (AIC) and Bayesian information criteria (BIC), and to the highest value of AUC (allsets command).

## 3. Results

A total number of 360 images were analyzed. Complete biofilm removal was not achieved in any abutment regardless of EA, MTD, and combination thereof. The ResB ranged from 26% to 48%.

### 3.1. Combination of MTD and EA

**Figure 3:**
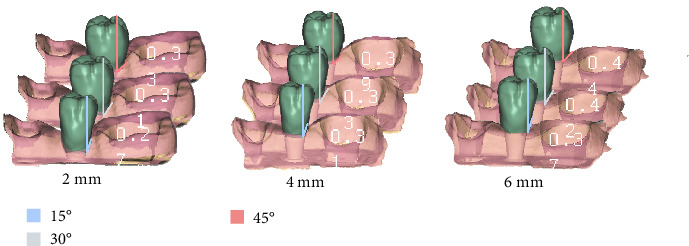
Residual biofilm (ResB) for the nine combinations of mucosal tunnel depth and emergence angle.

The mean ResB postintervention for each MTD-EA combination is shown in Figure 3. Higher ResB values were associated with increasing EA and MTD, showing a consistent upward trend. Intergroup comparisons revealed statistically significant differences (*p*  < 0.05) among most of the evaluated combinations. The configuration with a 6 mm MTD and a 45° EA exhibited the highest proportion of residual staining, showing significant differences compared to all other combinations (*p*  < 0.05). Conversely, the greatest extent of mechanical biofilm removal was observed on the abutment surface with the shallowest mucosal tunnel (2 mm) and a flattest emergence profile (15°).

### 3.2. MTD and EA by Surface Area

**Figure 4:**
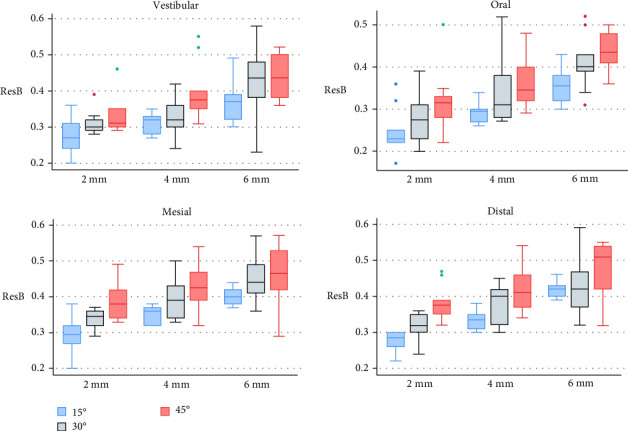
Residual biofilm (ResB) according to the four surfaces, for all mucosal tunnel depth and emergence angle combinations.

ResB results by surface area (vestibular, oral, mesial, and distal) are displayed in Figure 4. Interproximal surfaces consistently showed higher amounts of ResB across all combinations of EA and MTD. Taking into account the two variables independently, the amount of ResB consistently increases with MT depth, irrespective of the surface analyzed. Conversely, when focusing on EA, the data show that interproximal surfaces are more influenced by prosthesis shape than oral and vestibular surfaces (Table 1).

### 3.3. Logistic Regression Models

The predictive model presented in Table 2 identified both MTD and EA as significant predictors of ResB retention. The continuous outcome variable (ResB) was dichotomized based on its median value (0.31), resulting in a binary variable where abutments with ResB ≥0.31 were classified as having higher ResB after mechanical debridement.

Abutments with an MTD of 4 and 6 mm showed significantly higher odds of ResB compared to 2 mm, with odds ratios (ORs) of 7.15 and 65.44, respectively. Similarly, EAs of 30° and 45° were associated with higher ResB compared to 15°, with ORs of 4.20 and 13.77, respectively. However, the interaction term between MTD and EA was not statistically significant (*p*  > 0.05) (Table 2).

The best predictive model for the evaluated EA-MTD combinations is reported in Table 3. Among all configurations, the combination of an MTD of 6 mm and an EA of 45° emerged as the strongest predictor, with an OR of 134.33 (*p*  < 0.05), indicating a substantial increase in the likelihood of biofilm retention when both parameters are at their highest levels.

## 4. Discussion

The current in vitro experiment demonstrated that the prosthetic EA and the MTD have a statistically significant impact in terms of professional mechanical plaque removal efficacy. Remarkably, the complete removal of artificial biofilm from the abutment surfaces was never accomplished, irrespective of the EA and MTD. The intrinsic challenge of submucosal instrumentation mirrors that of nonsurgical subgingival treatment, namely, the lack of direct visibility during the procedure. The efficacy of nonsurgical instrumentation is well established, yet in vivo studies have demonstrated that achieving complete debridement of the root surface is unlikely [27]. However, it is known that a residual amount of plaque may persist that is nonetheless compatible with the re-establishment of periodontal health [28]. In the context of dental implants, it is recognized that the treatment of PIM has a lower probability of success compared to gingivitis, which can be attributed to the anatomical and prosthetic characteristics of the peri-implant environment. However, the percentage of ResB steadily increased with rising EA and MTD values. Moreover, less efficacy was observed across interproximal surfaces (M, D) compared to vestibular and oral sites. The logistic regression model confirmed the strong association between increased MTDs and wider prosthetic EAs with reduced efficacy of submucosal debridement. The decision to use the median value of ResB as the cut-off for the independent variable in the logistic regression is statistically motivated, as it enhances the identification of prosthetic features most likely to influence the study outcome. Furthermore, this analytical approach facilitates a more accessible interpretation of the results for a broader audience of professionals.

Previous clinical and in vitro investigations have assessed the role of MTD on peri-implant tissue health and biofilm removal. In a case-control study by Chan et al. [21], results showed that deep mucosal tunnel (≥3 mm) delays the resolution of inflammation, making it necessary to remove the crown and perform a submucosal professional cleaning to revert the inflammation at the baseline values. The impact of MTD on cleaning efficiency was brought up also by an in vitro study by Discepoli et al. [26], proving how the ResB statistically increases along with the MTD height, regardless the adjunctive use of glycine-powder air-polishing. Hence, such findings align with the present results, corroborating the idea that a suboptimal mucosal tunnel significantly affects treatment efficacy, hampering the accomplishment of PIM resolution.

Similar affirmations can be made regarding EA. A recent preclinical study conducted by Strauss et al. [19, 24] shed light upon the negative impact of a wide restorative angle on the integrity of the junctional epithelium and the marginal bone loss, marking the path for further analysis concerning EA clinical implications. Moreover, Katafuchi et al. [18] identified a higher incidence of peri-implantitis associated with the combination of a convex implant profile and a prosthetic EA greater than 30°. Similar findings were reported by Yi et al. [23], who observed a significantly greater marginal bone loss and higher prevalence of peri-implantitis in the presence of EAs equal to or greater than 30° (EA ≥ 30°) compared to those below 30°. Also, the interrelationship between the crown EA and the underlying abutment configuration is central to accessibility and biofilm retention. While evidence suggests that the restoration emergence profile, particularly coronal to the mucosal margin, exerts the greatest influence on cleaning efficacy [25, 29, 30], the abutment configuration is most relevant in the submucosal region, where convex contours or short abutments (<2 mm) may further compromise debridement efficacy and contribute to marginal bone loss, especially in the presence of a deep mucosal tunnel [31, 32].

Tuna et al. [33] investigated a modified prosthetic design that demonstrated enhanced cleaning efficacy in an in vitro model. Specifically, the implant replacing a mandibular molar was deliberately positioned along the axis of the distal root, rather than at the center of the edentulous area. This resulted in a narrow and precisely defined tunnel between the implant and the mesial pontic, which improved accessibility, promoting a more effective biofilm removal. These recent findings emphasize the clinician's strategic role in preventing peri-implant biological complications as early as the initial stages of surgical planning (primordial prevention) [3, 17–19, 23]. The establishment of the soft tissue barrier around dental implants is indeed influenced by both the mesiodistal and apicocoronal implant positioning [34, 35] and eventually shaped by the final prosthetic configuration [33]. While this study focused on MTD and EA, other prosthetic features such as prosthesis type (fixed vs. removable), retention mode (cemented versus screw-retained), crown contour, abutment connection, cantilever length, interproximal contact level, and open contacts have also been associated with differences in biofilm retention and peri-implant disease risk [36]. An increasing body of evidence is corroborating the emerging necessity of evaluating the implant-abutment-prosthesis complex, making its features as collectively determinant factors to clinical outcomes [17]. In this context, De Tapia et al. [14] investigated the treatment of PIM, focusing not only on the submucosal debridement, but also on the modification of prosthetic contours. The sole professional biofilm removal resulted in a 9.6% resolution rate, whereas patients who additionally received prosthetic correction achieved a significantly higher disease resolution amounting to 66.6%. Similar findings were obtained at the 30-month follow-up re-evaluation, reiterating the adjunctive effect of prosthetic contour modification [15].

In the present study, professional mechanical biofilm removal was performed via a magnetostrictive device without any adjunctive procedure. This choice was made because, according to current knowledge, no instrumentation protocol demonstrated a superiority compared to others in the treatment of PIM [3, 11, 37]. Future studies may investigate the effectiveness of different devices in relation to different combinations of prosthetic morphologies and transmucosal tract depths.

Against this background, the current study emphasizes the impact of abutment-prosthetic characteristics in the frame of preventive measures, highlighting the importance of strategic considerations from the treatment plan stage as “primordial” interventions [3]. As such, primordial prevention should not be limited to patient-performed oral hygiene but should begin even before implant installation, with the prosthetic design of the mucosal tunnel and the emergence profile. Indeed, proper prosthetic contours and adequate MTD may improve access to self-performed and professional plaque removal, enhancing the outcomes of peri-implant disease treatment and facilitating the long-term maintenance of therapeutic results within supportive peri-implant care (SPIC) programs [7].

### 4.1. Limitations

The present study faces some limitations within the design of an in vitro investigation. First, oral biofilm simulation using permanent ink may raise concerns regarding its applicability. As previously argued in earlier studies [38, 39], even though indelible ink undoubtedly exhibits physical properties distinct from those of bacterial plaque and may underestimate the true difficulty of biofilm removal from implant surfaces in clinical scenarios, the use of this technique confers several advantages. First, it allows for the creation of a surface that is easily analyzable and recognizable. Furthermore, it fully meets the objective of the study, namely, to demonstrate the feasibility of reaching the abutment surfaces in the transmucosal region via debridement devices in the absence of direct visualization. Therefore, the proof-of-concept presented in this study lies in the ability to clean a surface under various clinical conditions that might otherwise hinder the procedure.

Moreover, this in vitro model aims to replicate a clinical condition in a laboratory setting; however, a complete simulation of the access conditions and the physical and biological characteristics of the oral environment goes beyond the scope of the present study. Adjacent tissues (tongue and cheeks), the resistance of peri-implant mucosa, and the presence of biological fluids (saliva and crevicular fluid) influence operator performance. Moreover, peri-implant instrumentation can be performed using various devices (e.g., air-abrasive systems, PEEK/silicone tips, hand instruments), whose efficacy warrants further investigation in order to establish indications for their use in different clinical scenarios. Therefore, this study, while accounting for potential confounding factors, aims to investigate only a specific aspect of the complex oral and peri-implant system in order to support the rationale underlying the existing evidence, which identifies prosthetic configuration as a determining factor in the pathogenesis of peri-implant disease. Another limitation may be that the use of a single calibrated clinician who performed the instrumentation, combined with the absence of clinical limitations typically present in the oral environment, may have contributed to the consistency and robustness of the results. However, this design does not take into account the interoperator variability that inevitably occurs in clinical practice and may influence treatment outcomes in vivo.

Such limitations should be carefully considered when interpreting in vitro decontamination efficacy data and when extrapolating these findings to clinical practice.

## 5. Conclusions

Within the limitations of the present in vitro study, a progressive increase in the width of the prosthetic EA and the depth of the mucosal tunnel is associated with a consistent reduction of the submucosal debridement efficacy. In contrast, the association of narrower EA and shorter MTD yields beneficial results in terms of ResB. The interproximal surfaces consistently retain the highest levels of ResB, regardless of the EA and MTD. Complete removal of artificial biofilm was never achieved.

## Figures and Tables

**Table 1:** ANOVA testing ResB on different surfaces according to MT and EA.

MT	2 mm versus 4 mm		2 mm versus 6 mm		4 mm versus 6 mm	
	∆ (%)	*p*-Value	∆ (%)	*p*-Value	∆ (%)	*p*-Value
Vestibular	**4.3**	**0.041*⁣*^*∗*^**	**10.7**	**0.000*⁣*^*∗*^**	**6.4**	**0.001*⁣*^*∗*^**
Mesial	**5.1**	**0.004*⁣*^*∗*^**	**9.6**	**0.000*⁣*^*∗*^**	**4.5**	**0.014*⁣*^*∗*^**
Oral	**4.7**	**0.016*⁣*^*∗*^**	**11.9**	**0.000*⁣*^*∗*^**	**7.2**	**0.000*⁣*^*∗*^**
Distal	**5.4**	**0.004*⁣*^*∗*^**	**12.0**	**0.000*⁣*^*∗*^**	**6.5**	**0.000*⁣*^*∗*^**

**EA**	**15° versus 30°**		**15° versus 45°**		**30° versus 45°**	
	**∆ (%)**	***p*-Value**	**∆ (%)**	***p*-Value**	**∆ (%)**	***p*-Value**

Vestibular	3.7	0.171	7.0	**0.001*⁣*^*∗*^**	3.3	0.258
Mesial	**4.6**	**0.021** *⁣* ^*∗*^	**7.7**	**0.000*⁣*^*∗*^**	3.1	0.195
Oral	4.4	0.065	7.6	**0.000*⁣*^*∗*^**	3.1	0.323
Distal	3.5	0.178	8.6	**0.000*⁣*^*∗*^**	**5.1**	**0.024*⁣*^*∗*^**

*Note:* Bold values indicate statistical significance.

*⁣* ^*∗*^Bonferroni post hoc test corrected for multiple comparisons: *p*  < 0.05.

**Table 2:** Logistic regression model, MTD, EA, and MDT-EA interaction.

ResB ≥ 0.31						
LR chi^2^	Prob > chi^2^	Pseudo *R*^2^				
108.02	0.0000	0.27			**95% CI**	
**MTD**	**OR**	**SE**	***Z***	***p*-Value**	**Lower**	**Higher**

4 mm	7.15	3.62	3.88	**0.00**	2.64	19.33
6 mm	65.44	53.55	5.11	**0.00**	13.16	325.42
EA						
30°	4.20	2.08	2.91	**0.00**	1.59	11.09
45°	13.77	7.54	4.79	**0.00**	4.71	40.28
MDT#EA						
4 mm 30°	0.39	0.27	− 1.32	0.18	0.098	1.57
4 mm 45°	0.66	0.64	− 0.42	0.67	0.099	4.43
6 mm 30°	0.23	0.27	− 1.26	0.20	0.025	2.21

6 mm 45°	0.14	0.20	− 1.40	0.16	0.010	2.14

*Note*: MDT#EA, mucosal tunnel depth and emergence angle interaction terms. Bold values indicate statistical significance.

Abbreviations: EA, emergence angle; MTD, mucosal tunnel depth; ResB, residual biofilm.

**Table 3:** Logistic regression model, MTD-EA combinations.

ResB ≥ 0.31						
LR chi^2^	Prob > chi^2^	Pseudo *R*^2^				
104.57	0.0000	0.27			**95% CI**	
**MTD-EA**	**OR**	**SE**	***Z***	***p*-Value**	**Lower**	**Higher**

2 mm 30°	4.20	2.08	2.91	**0.00**	1.59	11.09
2 mm 45°	13.77	7.54	4.79	**0.00**	4.71	40.28
4 mm 15°	7.15	3.62	3.88	**0.00**	2.64	19.33
4 mm 30°	11.86	6.35	4.62	**0.00**	4.15	33.88
4 mm 45°	65.44	53.55	5.11	**0.00**	13.16	325.42
6 mm 15°	65.44	53.55	5.11	**0.00**	13.16	325.42
6 mm 30°	65.44	53.55	5.11	**0.00**	13.16	325.42
6 mm 45°	134.33	145.24	4.53	**0.00**	16.13	1118.17

*Note:* Bold values indicate statistical significance.

Abbreviations: MTD-EA, mucosal tunnel depth-emergence angle combinations; ResB, residual biofilm.

## Data Availability

The data sets used and/or analyzed during the current study are available from the corresponding author on reasonable request.

## Supporting Information

Supporting Information Figure S1. Residual biofilm (ResB) visualization through ImageJ.
