# Supplementary figures and images for: Influence of Emergence Angle and Mucosal Tunnel Depth on Artificial Biofilm Removal Around Dental Implants: An In Vitro Study
Source: Int J Dent. 2025 Nov 11;2025:7500003. doi: 10.1155/ijod/7500003 (PMC12626696; doi:10.1155/ijod/7500003)

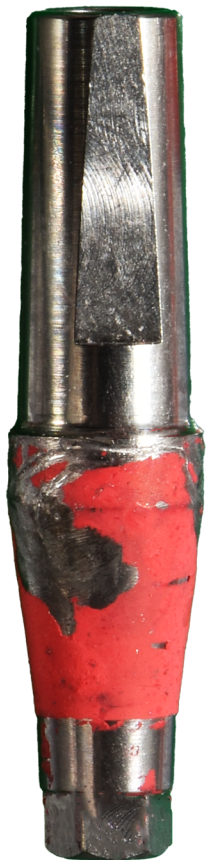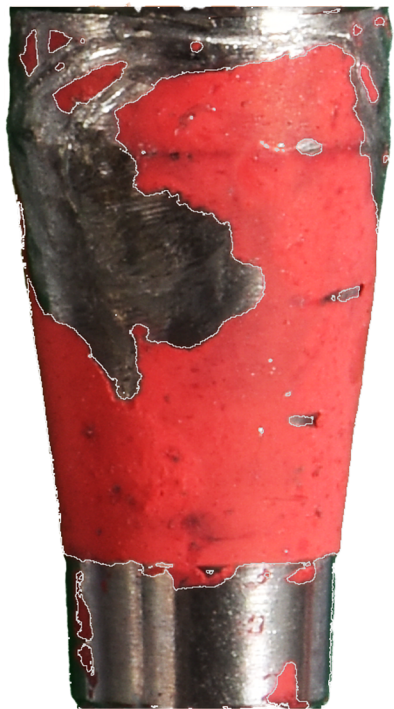

Supplement: Supporting Information — Figure S1. Residual biofilm (ResB) visualization through ImageJ. [file 7500003.f1.pdf]
